# Robust Polyurethane Hydrogels Based on Dynamic Disulfide Bonds and Pendant Tertiary Amines with Room-Temperature Self-Healing and pH Responsiveness

**DOI:** 10.3390/gels12060555

**Published:** 2026-06-20

**Authors:** Xia Ding, Bing Yang, Xinyi Si, Lei Ni, Chao Fang, Zhaosheng Hou

**Affiliations:** 1 School of Intelligence Engineering, Shandong Management University, Jinan 250357, China; 14438120160212@sdmu.edu.cn (X.D.); nilch@163.com (L.N.); 2 College of Chemistry, Chemical Engineering and Materials Science, Shandong Normal University, Jinan 250014, China; 202410100512@stu.sdnu.edu.cn (X.S.); fangchao@sdnu.edu.cn (C.F.)

**Keywords:** polyurethane hydrogel, dynamic disulfide bonds, tertiary amines, pH responsiveness, self-healing, mechanical properties

## Abstract

Hydrogels have garnered significant attention due to their tunable structures and broad applicability in biomedical and smart materials. However, achieving a balance between excellent mechanical performance and multifunctionality remains a major challenge. In this study, a series of multifunctional polyurethane hydrogels (PUGs) was developed by integrating dynamic disulfide bonds and pendant tertiary amine groups into poly(ethylene glycol)-based networks using a solvent-exchange method. Structural characterization confirmed the successful formation of a crosslinked porous network. The hydrogels demonstrated remarkable mechanical properties, with PUG–II exhibiting a tensile strength of 448 kPa and an elongation at break of 489%, as well as exceptional compressibility (371 kPa at 90% strain) and fatigue resistance. Meanwhile, the PUGs displayed efficient room-temperature self-healing with a healing efficiency of up to 94.5%. The reversible protonation of tertiary amine groups imparted pronounced pH-responsive swelling behavior, with the equilibrium swelling ratio of PUG–I at pH 2.0 being 5.8 times higher than that at pH 12.0. This study provides a promising strategy for developing PU-based hydrogels that combine robust mechanical performance and multifunctionality, offering potential for advanced smart material applications.

## 1. Introduction

Hydrogels are three-dimensional crosslinked polymer networks composed of hydrophilic macromolecules. Their high water content, porous structure, and tunable physicochemical properties closely resemble those of biological soft tissues, rendering them highly attractive for biomedical applications [1]. Hydrogels are commonly classified into natural and synthetic types. Natural hydrogels include collagen, chitosan, hyaluronic acid, and dextran [2,3,4,5], whereas synthetic hydrogels are typically represented by polyethylene glycol (PEG), poly(acrylic acid), and polyacrylamide [6,7,8]. Although natural hydrogels exhibit excellent biocompatibility, biodegradability, and intrinsic bioactivity, they usually suffer from limited mechanical strength [9,10]. In contrast, synthetic hydrogels can be rationally engineered via the incorporation of functional components or nanomaterials, enabling enhanced mechanical performance, electrical conductivity, and biological functionality, as well as precise control of degradation and swelling behavior.

Polyurethanes (PUs), owing to their excellent flexibility, biocompatibility, and highly tunable structure, have emerged as a versatile platform for hydrogel fabrication [11]. PU-based hydrogels (PUGs) exhibit adjustable mechanical properties while maintaining the inherent water-retention capability of conventional hydrogels [12,13]. By integrating the mechanical robustness of PU with the hydrophilicity of polymer networks, PUGs have demonstrated broad potential in tissue engineering, drug delivery, wound dressings, and smart materials [14]. Nevertheless, conventional PUGs still face two critical challenges in practical applications. First, mechanical damage such as microcracks or macroscopic fractures is unavoidable during service, and the lack of self-repair capability leads to performance deterioration and reduced service lifespan. Second, the high water content weakens intermolecular interactions, resulting in insufficient mechanical strength for load-bearing applications [15,16,17]. Therefore, developing PUGs that simultaneously exhibit high mechanical strength and efficient autonomous self-healing capability without compromising hydration remains a key challenge [18].

To address these issues, dynamic covalent bonds have been widely introduced into polymer networks to impart self-healing capability [19,20,21]. Among them, disulfide bonds are particularly attractive due to their reversible cleavage and reformation under mild conditions, such as thermal or ultraviolet stimuli, enabling repeated healing of damaged structures [22,23]. Previous studies have demonstrated that incorporating disulfide bonds into PU systems can effectively endow materials with self-healing properties while maintaining structural integrity [24]. However, the exclusive reliance on disulfide exchange often leads to a trade-off between healing efficiency and mechanical strength, as dynamic bonds may reduce network stability and cause mechanical deterioration upon swelling [25]. Therefore, alternative molecular design strategies are required to achieve a better balance between strength and healing performance.

Tertiary amine groups play a multifunctional role in PU-based systems. They can act as catalysts to regulate polyurethane gelation [26,27] and can also enhance intermolecular interactions through electrostatic effects by forming quaternary ammonium species, thereby imparting pH responsiveness [28,29]. More importantly, tertiary amines serve as hydrogen bond acceptors, forming extensive hydrogen bonding with urethane groups or water molecules. These reversible physical interactions facilitate energy dissipation under external stress, thereby improving mechanical strength and toughness [30,31]. Consequently, the incorporation of tertiary amine groups is expected to introduce multiple noncovalent interactions that synergistically reinforce the dynamic covalent network.

In this work, a synergistic design strategy was proposed by integrating disulfide bonds and tertiary amine groups into PU-based hydrogels. PEG-based PU prepolymers containing pendant tertiary amine groups were synthesized, followed by the introduction of disulfide-containing extenders/crosslinkers to construct a hybrid network featuring both dynamic covalent crosslinking and multiple physical interactions. The resulting PUGs were prepared via a solvent-exchange method. In contrast to previously reported PU hydrogels that mainly rely on either disulfide exchange for self-healing or ionizable groups for pH-responsive swelling [32,33], the present system combines these two functional motifs within one network. Dynamic disulfide bonds enable interfacial reconstruction after damage, while pendant tertiary amine groups provide reversible hydrogen bonding and pH-sensitive protonation sites, thereby contributing to energy dissipation, network stability, and pH-dependent swelling. The effects of disulfide content and tertiary amine structure on mechanical properties, pH-responsive swelling behavior, and self-healing performance were systematically investigated. Furthermore, the synergistic mechanism between dynamic covalent bonds and noncovalent interactions was elucidated. This work provides a feasible strategy for designing multifunctional PU hydrogels with enhanced toughness, pH responsiveness, and self-healing capability.

## 2. Results and Discussion

### 2.1. FTIR Spectra

**Figure 1:**
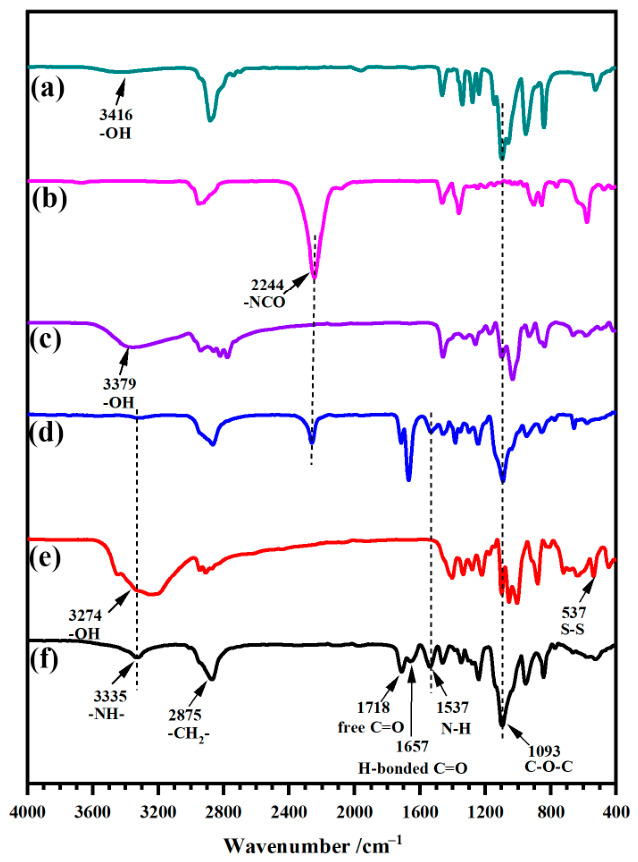
FTIR spectra of (**a**) PEG, (**b**) IPDI, (**c**) DAP, (**d**) PUP (DMF solution), (**e**) DSO, and (**f**) DPUG–II.

The FTIR spectra of PEG, IPDI, DAP, PUP (DMF solution), DSO, and the representative dried hydrogel DPUG–II are shown in Figure 1. In the spectrum of PUP (Figure 1d), the broad –OH stretching bands of PEG (3416 cm^−1^, Figure 1a) and DAP (3379 cm^−1^, Figure 1c) disappeared, accompanied by the emergence of a new peak at 3335 cm^−1^, corresponding to the –NH– stretching vibration of urethane linkages. This observation confirmed the successful formation of urethane bonds. In addition, compared with the spectrum of IPDI (Figure 1b), the characteristic –NCO absorption band at 2244 cm^−1^ exhibited a pronounced decrease in intensity, indicating that most –NCO groups were consumed during the reaction, while only a minor fraction remained at the termini of the PUP chains. For DPUG–II (Figure 1f), the complete disappearance of the –NCO peak demonstrated that all residual –NCO groups reacted with –OH groups from DSO. Meanwhile, the absence of the broad –OH band of DSO at 3274 cm^−1^ (Figure 1e) further confirmed the complete consumption of –OH groups, suggesting that DSO fully participated in the crosslinking process. Characteristic absorption bands at 3335, 1718, 1657, and 1537 cm^−1^ were assigned to urethane –NH– stretching, free C=O stretching, hydrogen-bonded C=O stretching, and N–H bending vibrations [34], respectively, all of which exhibited increased intensities compared with those of PUP. The ether C–O–C stretching vibration of the soft segment appeared at 1093 cm^−1^, while the S–S vibration from the disulfide bonds was weakly detectable at 537 cm^−1^ due to its low content. These spectral features verified the successful formation of the crosslinked DPUG network.

### 2.2. XPS Spectra

**Figure 2:**
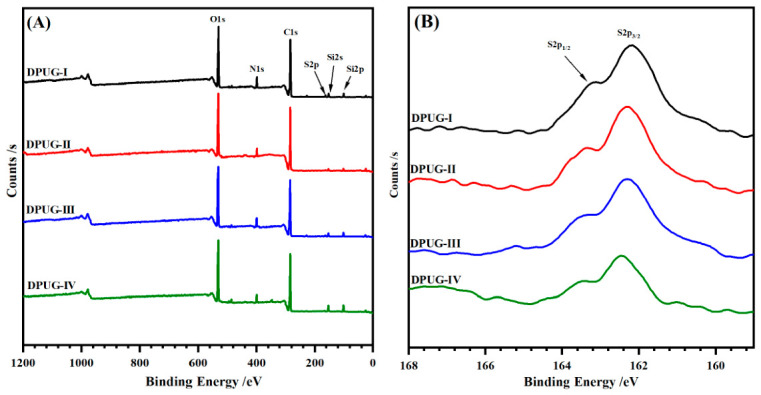
(**A**) Wide-scan and (**B**) fine-scan XPS spectra of DPUGs.

The wide-scan XPS spectra of the DPUGs are presented in Figure 2A. Distinct peaks located at 531.7, 397.3, and 283.8 eV were assigned to O 1s, N 1s, and C 1s, respectively. A weak peak at approximately 162 eV was attributed to the S 2p signal of disulfide bonds derived from DSO. The low intensity of this peak was consistent with the relatively low sulfur content in the materials. Fine-scan spectra of the S 2p region (Figure 2B) revealed two characteristic peaks at 163.3 eV (S 2p_1_/_2_) and 162.2 eV (S 2p_3_/_2_) [35]. The intensity of these peaks gradually decreased from DPUG–I to DPUG–IV, consistent with the reduction in sulfur content. Signals observed at 149.1 eV (Si 2s) and 100.6 eV (Si 2p) were attributed to trace silicon contamination introduced from glassware during sample preparation. These results provided further evidence for the successful incorporation of disulfide bonds into the polymer network.

### 2.3. Micromorphology

**Figure 3:**
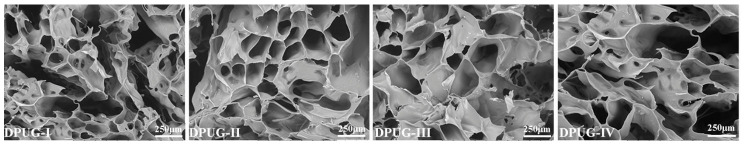
SEM images of DPUGs.

The micromorphology of hydrogels is intrinsically related to their mechanical performance, and a homogeneous and dense network structure is generally essential for achieving superior mechanical properties [36]. The microstructures of DPUGs were characterized by SEM, as depicted in Figure 3. All DPUG samples displayed a porous, honeycomb-like network morphology, with pore size increasing progressively from DPUG–I to DPUG–IV. To quantitatively evaluate the pore dimensions, twenty pores were randomly selected from each SEM image. Pore size measurements and pore size distribution analysis were performed using Photoshop CS6 and Origin 2021, respectively. The average pore sizes of DPUG–I, –II, –III, and –IV were 149 ± 8.2, 192 ± 11.3, 241 ± 14.0, and 277 ± 16.1 μm, respectively. Given the comparable solid contents of the PUGs, pore size was mainly governed by the crosslinking density. Increasing the molecular weight of PEG raised its proportion in the network while reducing the relative content of DSO crosslinker, resulting in decreased crosslinking density and larger pore structures. Despite this trend, all DPUGs maintained a relatively compact and interconnected porous network, which facilitated efficient dissipation of applied stress and contributed to enhanced tensile and compressive strength. In addition, the abundant interconnected porous structure provided sufficient free volume for water retention, indicating the excellent water-holding capacity of the hydrogels.

### 2.4. Thermal Stability

**Figure 4:**
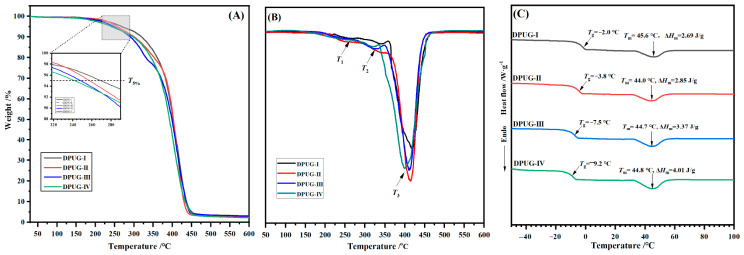
(**A**) TGA, (**B**) DTGA, and (**C**) DSC curves of DPUGs.

The thermal stability of the DPUGs was evaluated by TGA, and the corresponding TGA and DTGA curves are presented in Figure 4A and Figure 4B, respectively. As shown in Figure 4A, all samples exhibited negligible weight loss below 200 °C, indicating the absence of residual solvents and/or unreacted monomers in the materials. As the PEG molecular weight increased from DPUG–I to DPUG–IV, the temperature corresponding to 5% weight loss (*T*_5_%) progressively shifted to lower values. This trend could be attributed to the reduced crosslinking density, which weakened intermolecular interactions [37]. DTGA analysis (Figure 4B) revealed that the thermal degradation of DPUGs mainly proceeded through three stages. Considering that PU networks contain several thermally degradable structural units, their decomposition generally involves partially overlapping processes rather than completely independent steps [38]. Therefore, the degradation stages observed in the DTGA curves were cautiously interpreted based on the peak positions and the chemical structure of the DPUGs. The first stage, with a maximum decomposition temperature (*T*_1_) around 250 °C, could be mainly related to the cleavage of thermally labile disulfide bonds, together with the initial degradation of urethane-containing hard segments. The relatively small mass loss in this stage was consistent with the low content of disulfide bonds in the network. The second stage, with *T*_2_ at approximately 320 °C, may be primarily associated with further decomposition of urethane linkages and hard-segment structures. The third stage, with *T*_3_ near 410 °C, was likely dominated by the pyrolysis of PEG-based ether soft segments, accompanied by the further decomposition of residual polymer fragments. All samples exhibited a residual mass of less than 3 *wt*% at 600 °C, indicating nearly complete thermal decomposition. The minor residue may originate from inorganic residues from the tin catalysts.

### 2.5. Thermal Transition

The DSC curves of the DPUGs and the corresponding thermal transition parameters are shown in Figure 4C. All samples exhibited a single glass transition temperature (*T*_g_), indicating good component compatibility and the absence of evident microphase separation. With increasing PEG molecular weight, the *T*_g_ values of DPUG–I to DPUG–IV decreased progressively from −2.0 to −3.8, −7.5, and −9.2 °C, respectively. This trend could be mainly attributed to the reduced crosslinking density, which enhanced the segmental mobility of the polymer chains [39]. Additionally, the increased content of flexible PEG segments further contributed to the decrease in *T*_g_. Since all *T*_g_ values were well below ambient temperature, the corresponding hydrogels remained in a rubbery state under ambient conditions. The enhanced chain mobility also facilitated the dynamic exchange of disulfide bonds, thereby promoting the self-healing capability of the hydrogels. Furthermore, all samples displayed a weak endothermic melting peak at approximately 45 °C (*T*_m_), which was assigned to the melting of crystalline PEG soft segments. As the crosslinking density decreased from DPUG–I to DPUG–IV, the corresponding melting enthalpy (Δ*H*_m_) increased gradually from 2.69 to 4.01 J/g. This behavior was ascribed to two factors: first, the reduced crosslinking density improved chain mobility, facilitating the rearrangement of soft segments and thus enhancing crystallinity [40]; second, the content of crystallizable PEG segments within the polymer network increased. However, the low Δ*H*ₘ values (<5.0 J/g) indicated limited crystallinity, suggesting that the DPUGs were predominantly amorphous.

### 2.6. pH-Dependent Swelling Behavior

**Figure 5:**
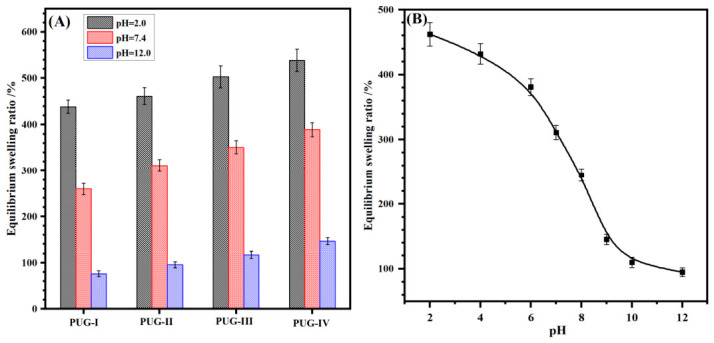
Equilibrium swelling ratios of (**A**) PUGs at various pH values and (**B**) PUG–II over a narrow pH range.

According to previous reports [41], PU elastomers containing pendant tertiary amine groups typically exhibit pronounced pH-responsive swelling behavior. Therefore, the fabricated PUGs are expected to display similar pH sensitivity. The equilibrium swelling ratios (ESRs) of PUGs under different pH conditions are presented in Figure 5A. As expected, all samples exhibited distinct pH-dependent swelling behavior, with higher ESR values under acidic conditions and lower values under alkaline environments. Furthermore, as the tertiary amine content increased from PUG–IV to PUG–I, the disparity in ESR between acidic and alkaline environments became more pronounced. For instance, in PUG–I (with the highest tertiary amine content), the ESR at pH 2.0 was approximately 1.7 and 5.8 times higher than those at pH 7.4 and 12.0, respectively. In contrast, these ratios for PUG–IV with the lowest tertiary amine content decreased to 1.4 and 3.7. This pH-responsive swelling behavior is mainly governed by the reversible protonation/deprotonation of pendant tertiary amine groups: under acidic conditions, especially when the external pH is far below the pKa of tertiary amine groups, tertiary amines are extensively protonated into positively charged ammonium groups. The generated fixed positive charges increase electrostatic repulsion among polymer chains and induce osmotic pressure due to mobile counterions, thereby driving more water into the network and resulting in significantly enhanced swelling [42,43]. Under alkaline conditions, tertiary amine groups are mostly deprotonated, and hydrogen bonding between tertiary amine and urethane moieties becomes dominant, leading to a more compact network and reduced swelling. Notably, although the tertiary amine content decreased from PUG–I to PUG–IV, the ESR values under all pH conditions showed an overall increasing trend. This phenomenon could be ascribed to the increased content of hydrophilic PEG segments and the reduced crosslinking density within the polymer network, both of which promote water absorption and counteract the effect of the decreased tertiary amine content. To further investigate the influence of external pH on swelling behavior, PUG–II was selected as a representative sample, and its ESR was measured over a narrower pH range (Figure 5B). A sharp transition in ESR was observed between pH 9 and 10, which was close to the pKa value (~9.5) of the alkyl tertiary amine groups [44]. Around this pH range, a slight change in external pH can cause a rapid change in the protonation degree of tertiary amine groups, leading to an abrupt variation in electrostatic repulsion and osmotic pressure within the network. Therefore, the sharp ESR transition at pH 9–10 originates from the protonation/deprotonation equilibrium of tertiary amine groups near their pKa value. The result further confirms that the remarkable swelling increase under acidic conditions originates from the protonation of tertiary amine groups. Because this protonation/deprotonation process is reversible, the hydrogel network is expected to undergo reversible swelling/deswelling when the environmental pH alternates between acidic and alkaline conditions. This pronounced transition suggests that the PUGs possess promising potential as reversible pH-responsive smart materials.

### 2.7. Mechanical Properties

**Figure 6:**
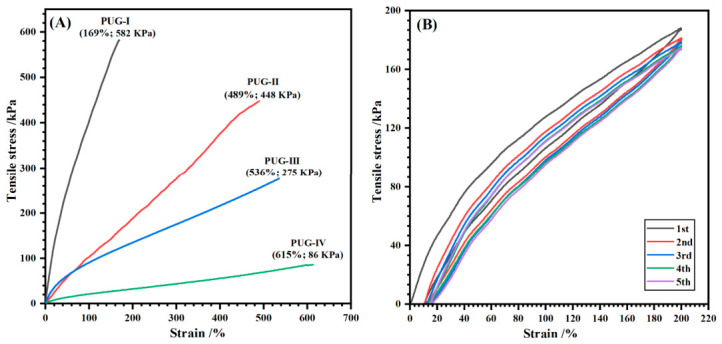
(**A**) Tensile curves of PUGs and (**B**) consecutive cyclic tensile curves of PUG–II at 200% strain.

Uniaxial tension: The uniaxial tensile stress–strain curves of the PUGs are depicted in Figure 6A. All samples exhibited nearly linear stress–strain behavior without a distinct yielding point during stretching, indicating predominantly elastic deformation. As the molecular weight of PEG increased from PUG–I to PUG–IV, the maximum tensile strength (σ_t−m_) gradually decreased, whereas the elongation at break (ε_t−m_) increased. This trend was primarily attributed to the increased content of flexible PEG segments and the corresponding decrease in crosslinking density within the network. Meanwhile, although the molar amount of DAP was fixed in the feed formulation, the calculated tertiary amine content decreased from PUG–I to PUG–IV with increasing PEG molecular weight. Tertiary amine groups can act as hydrogen-bond acceptors and form reversible interactions with urethane N–H groups and water molecules [45,46], thereby enhancing intermolecular cohesion and energy dissipation. Therefore, higher tertiary amine content is beneficial for improving the tensile strength, but may also restrict chain mobility and reduce extensibility. Accordingly, the tensile properties of the PUGs are governed by the combined effects of tertiary amine content, crosslinking density, and PEG chain flexibility. Although the tensile properties are influenced by multiple factors, the PUGs demonstrated a favorable balance of tensile strength and extensibility, which was superior to many reported PEG-based hydrogels [37,47]. Notably, PUG–II, featuring moderate PEG content, tertiary amine content, and crosslinking density, exhibited optimal overall tensile performance with σ_t−m_ and ε_t−m_ values of 448 kPa and 489%, respectively. High stretchability is particularly advantageous for self-healing systems, as it helps balance tensile strength and healing efficiency.

Cyclic tension: Cyclic tensile loading–unloading tests were performed using PUG–II as a representative sample because it exhibited the optimal overall tensile performance among the prepared hydrogels, with a favorable balance between tensile strength and stretchability. The corresponding stress–strain curves over five consecutive cycles at a fixed strain of 200% are shown in Figure 6B. Only minor hysteresis loops were observed during all cycles, indicating limited energy dissipation and good elastic recovery. As the cycle number increased, σ_t−m_ slightly decreased, accompanied by a minor increase in residual strain (εₜ). Nevertheless, after five cycles, the hydrogel still retained a high stress retention ratio (σ_t−5_/σ_t−1_) of 92.7% and exhibited a low residual strain of 15.9%, demonstrating excellent resistance to tensile fatigue.

**Figure 7:**
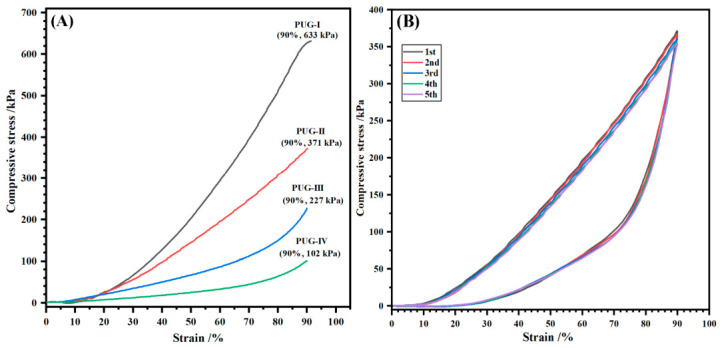
(**A**) Compressive curves of PUGs at 90% strain and (**B**) consecutive cyclic compressive curves of PUG–II at 90% strain.

Uniaxial compression: Figure 7A illustrates the uniaxial compressive stress–strain curves of the PUGs at a maximum compressive strain of 90%, which was selected to avoid instrument damage. The maximum compressive strength (σ_c−m_) decreased from 633 kPa (PUG–I) to 86 kPa (PUG–IV). Given the similar solid contents, this trend was mainly governed by crosslinking density. Lower crosslinking density leads to larger pore sizes and a looser network structure, thereby reducing compressive strength. Nevertheless, even PUG–IV, which had the lowest crosslinking density among the samples, exhibited a σ_c−m_ value of 86 kPa, demonstrating considerable compressive robustness. Importantly, all samples withstood compressive strains up to 90% without structural failure, highlighting their remarkable toughness.

Cyclic compression: Cyclic compression tests were further conducted on PUG–II, and the corresponding curves are presented in Figure 7B. The compressive stress–strain curves from five consecutive cycles almost completely overlapped, indicating negligible energy dissipation [48]. This behavior suggested that the hydrogels could rapidly recover their original shape upon unloading, demonstrating outstanding resistance to compressive fatigue.

**Figure 8:**
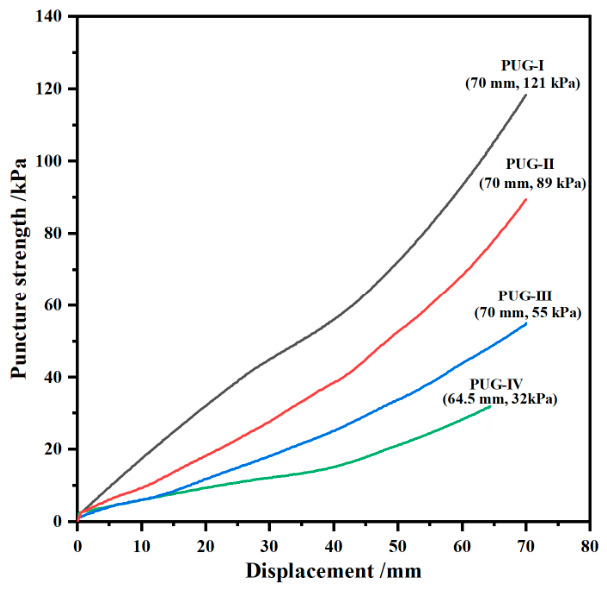
Puncture resistance load–displacement curves of the PUGs at 70 mm displacement.

Puncture resistance: The representative puncture load–displacement curves of the PUGs are shown in Figure 8 (with a maximum displacement limited to 70 mm to prevent instrument damage). The puncture strength increased progressively with displacement, exhibiting an approximately linear relationship. At the maximum displacement, all samples except PUG–IV (with the lowest crosslinking density) remained unpenetrated. The maximum puncture strengths for PUG–I, –II, –III, and –IV were 121, 89, 55, and 32 kPa, respectively. These results indicated that higher crosslinking density significantly enhanced the puncture resistance of the hydrogels.

### 2.8. Self-Healing Properties

**Figure 9:**
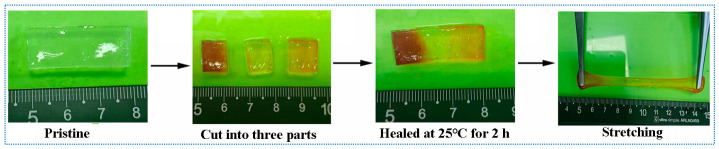
Images of the cutting–healing–stretching procedure of PUG–II.

Qualitative evaluation: The self-healing behavior of the PUG–II hydrogel was first qualitatively evaluated, as illustrated in Figure 9. The hydrogel was cut into three pieces, which were then carefully brought into contact and allowed to heal at room temperature. The cut interfaces gradually vanished and became indistinguishable to the naked eye, accompanied by evident dye diffusion across the interfaces, indicating molecular interdiffusion and network reconstruction. After healing for 2 h at room temperature, the rejoined sample was subjected to manual stretching. The healed hydrogel could be stretched up to approximately three times its original length without fracture, demonstrating effective restoration of mechanical integrity. These observations confirmed that the dynamic network enabled efficient reconnection of fractured interfaces [49]. The rapid disappearance of the interface and recovery of stretchability suggested that dynamic covalent interactions and chain mobility played a crucial role in facilitating the self-healing process [50]. Moreover, hydrogen bonding among urethane groups and tertiary amine groups may also play an auxiliary role in interfacial adhesion and network reconstruction during self-healing [51].

**Figure 10:**
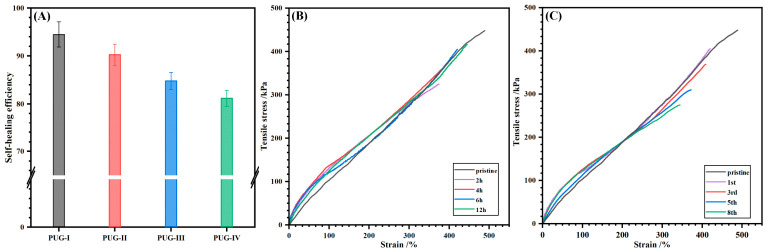
(**A**) Self-healing efficiency of PUGs healed at 25 °C for 6 h; (**B**) tensile stress–strain curves of PUG–II healed at 25 °C for various healing times; (**C**) tensile stress–strain curves of PUG–II healed at 25 °C for 6 h after multiple fracture-healing cycles.

Quantitative evaluation: The self-healing performance of the hydrogels under different conditions (healing time and repeated fracture-healing) was further quantitatively evaluated by the calculated healing efficiency (η). The η values of the PUGs after healing at room temperature for 6 h are summarized in Figure 10A. It was evident that η decreased progressively with decreasing DSO content, indicating that the self-healing capability primarily originated from the reversible exchange of disulfide bonds. Notably, PUG–I, which contained the highest disulfide bond content, exhibited a high η value of 94.5% after healing for 6 h at room temperature, demonstrating excellent intrinsic room-temperature self-healing ability. Taking PUG–II as a representative sample, the effects of healing time and repeated healing cycles on η were further investigated, as shown in Figure 10B and Figure 10C, respectively. As the healing time increased from 2 h to 6 h, η increased rapidly from 72.5% to 90.2%, while further extension of the healing time to 12 h resulted in only a slight increase to 92.8%. This indicated that the self-healing process was largely completed within the initial 6 h. The tensile stress–strain curves of the healed samples at different healing times remained nearly linear, suggesting effective network reconstruction. As expected, both σ_t−m_ and ε_t−m_ gradually decreased with increasing healing cycles. The η values of PUG–II after the 1st, 3rd, 5th, and 8th healing cycles were 90.2%, 82.2%, 68.9%, and 61.2%, respectively. This decline could be attributed to the accumulation of irreversible structural damage during repeated fracture-healing processes, such as chain scission or incomplete network reformation. Nevertheless, even after eight cycles, the hydrogel maintained an η value above 60%, highlighting its robust and repeatable self-healing capability.

## 3. Conclusions

In this work, a series of PUGs was successfully developed by integrating dynamic disulfide bonds and tertiary amine groups, achieving a combination of superior mechanical properties and multifunctionality. Structural analyses confirmed the formation of a stable crosslinked network with a homogeneous porous morphology. The PUGs exhibited excellent tensile and compressive performance and good fatigue resistance. PUG–II demonstrated a σ_t−m_ of 448 kPa, an ε_t−m_ of 489%, and σ_c−m_ of 371 kPa at 90% strain. The pendant tertiary amine groups endowed the hydrogels with pronounced pH-responsive swelling behavior, with the ESR of PUG–I at pH 2.0 being 5.8 times higher than that at pH 12.0. Dynamic disulfide exchange enabled efficient room-temperature self-healing, with an η of up to 94.5%. Quantitative analysis of crosslinking density will be further conducted in future work to better clarify the structure–property relationships. This synergistic strategy provides a feasible and effective approach for developing mechanically robust, pH-responsive, and self-healing PU-based hydrogels for advanced applications.

## 4. Materials and Methods

### 4.1. Materials

3-Mercapto-1,2-propanediol (MPD, 97%), PEG (M_n_ = 0.6, 1.0, 1.5, and 2.0 kDa), Stannous octoate ((Oct)_2_Sn, >95%), and isophorone diisocyanate (IPDI, 99%) were purchased from Macklin (Shanghai, China). Dimethylsulfoxide (DMSO, GR) and anhydrous N,N-dimethylformamide (DMF, moisture content < 0.05%) were obtained from Sigma-Aldrich (Shanghai, China). 3-Dimethylamino-1,2-propanediol (DAP, ≥98%), dichloromethane (DCM, AR), and rhodamine B (CP) were supplied by Aladdin Reagent Co., Ltd. (Shanghai, China). Other chemicals were used as received.

### 4.2. Synthesis of 3,3′-Dithiodipropane-1,2-diol (DSO)

**Figure 11:**
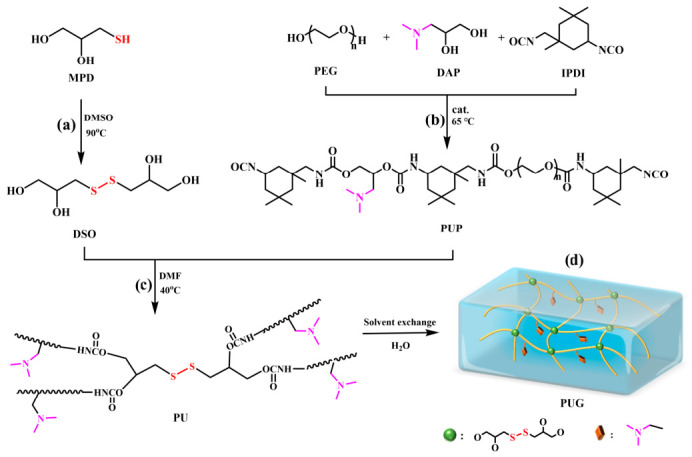
Synthetic routes of (**a**) DSO, (**b**) PUP, and (**c**) PU; (**d**) structural schematic diagrams of hydrogels.

DSO was synthesized according to the route shown in Figure 11a and our previously reported method [25]. Briefly, MPD (11.2 g, 100 mmol) was dissolved in 20 mL of DMSO, and the reaction was carried out at 90 °C under stirring for 12 h. The resulting solution was slowly added dropwise into 200 mL of precooled DCM (0 °C) under mechanical stirring and allowed to stand for 30 min. The precipitate was collected by vacuum filtration, washed three times with cold DCM, and dried under vacuum to constant weight. The obtained product (DSO) was a white powder with a yield of 89.3%.

^1^H NMR (400 MHz, ppm, DMSO–d_6_, Figure S1): δ 2.58~2.97 (m, 4H, S–CH_2_), 3.38 (m, 2H, CH_2_–OH), 3.66 (m, 2H, –CH–), 4.64 (t, 2H, CH–OH), 4.88 (d, 2H, CH_2_–OH); ^13^C NMR (101 MHz, ppm, DMSO–d_6_, Figure S2): δ 43.5 (CH_2_–S), 65.0 (CH–OH), 70.7 (CH_2_–OH); MS (*m*/*z*, Figure S3) calculated for C_6_H_14_O_4_S_2_ [M + Na^+^] 237.0226, found 237.0217.

### 4.3. Synthesis of PUs and Preparation of PUGs

Based on the compositions listed in Table 1, DAP and dehydrated PEG were first dissolved in anhydrous DMF. IPDI and (Oct)_2_Sn (0.25 wt% relative to the total monomers) were then added into the solution. The reaction was carried out at 65 °C under a nitrogen atmosphere for approximately 50 min until the –NCO content reached the theoretical value, as determined by the dibutylamine titration method. Subsequently, the reaction mixture was diluted with an appropriate amount of anhydrous DMF, gently stirred, and cooled to 40 °C. A DSO solution in DMF (0.1 g/mL) was then added, followed by further stirring for 5 min. After degassing under reduced pressure, the solution was slowly cast into a PTFE mold and sealed. The sample was cured at 40 °C for approximately 24 h to obtain PU gels. The PU gels were immersed in deionized water for 7 days, during which the water was refreshed every 24 h, yielding PU hydrogels (PUGs). The synthetic routes and structural schematics are illustrated in Figure 11b–d. The hydrogels were denoted as PUG–I, –II, –III, and –IV according to the increasing PEG molecular weight (0.6–2.0 kDa). The corresponding freeze-dried samples were referred to as DPUGs.

### 4.4. Instruments and Characterization

Structural characterization: ^1^H/^13^C NMR spectra were recorded on an AVANCE II 400 MHz spectrometer (Bruker, Ettlingen, Germany) using DMSO–d_6_ as the solvent. FTIR spectra were collected on an ALPHA II spectrometer (Bruker, Ettlingen, Germany) at room temperature in the range of 4000–400 cm^−1^. Mass spectra were obtained on an LCMS–2020 spectrometer (Shimadzu, Kyoto, Japan). XPS analysis was conducted using an ESCALAB 250Xi instrument (Thermo Scientific, Waltham, MA, USA) equipped with Al Kα radiation (1486.68 eV). Surface morphologies were observed by SEM (SU8010, Hitachi, Kyoto, Japan) after gold sputtering.

Thermal properties: TGA was carried out on a Q50 instrument (TA Instruments, New Castle, DE, USA) under nitrogen (35 mL/min) at a heating rate of 20 °C/min from room temperature to 600 °C. DSC (DSC 2910, TA Instruments, New Castle, DE, USA) was conducted from −40 to 100 °C at a heating rate of 10 °C/min under nitrogen (35 mL/min). All samples were preheated prior to testing to eliminate thermal history.

Mechanical Properties: Tensile tests were performed on an STMA 1.01 tester (Shanghai, China) at a crosshead speed of 20 mm/min using dog-bone-shaped specimens (width: 4 mm; gauge length: 30 mm). Compression tests were conducted on a CT3 texture analyzer (Brookfield Engineering Laboratories, Inc., Middleboro, MA, USA) with a TA10A probe. Cylindrical samples (D: 12.0 mm; H: 10.0 mm) were tested at room temperature with a compression rate of 0.2 mm/s up to 90% strain. Puncture resistance was measured on the same analyzer using a 5.0 mm probe. Hydrogel films (5 × 5 × 0.3 cm^3^) were tested at 50 mm/min with a maximum displacement of 70 mm.

Swelling behavior: pH-responsive swelling was evaluated gravimetrically in buffer solutions (pH 2.0, 7.4, and 12.0) at room temperature. The swelling ratio was defined as the percentage mass increase relative to the initial weight. Measurements were continued until swelling equilibrium was reached.

Self-healing properties: For qualitative evaluation, hydrogel strips were cut into sections, and selected pieces were stained using rhodamine B. The cut surfaces were brought into contact and allowed to heal at room temperature under 50% relative humidity. The self-healing process was monitored by dye diffusion and manual stretching. For quantitative analysis, dog-bone-shaped specimens were cut at the midpoint and rejoined without external force. After healing under predefined conditions, tensile tests were conducted. The self-healing efficiency (η) was calculated as the ratio of the tensile strength of healed samples to that of the original samples.

All experiments were performed in triplicate, and the quantitative data were presented as mean values ± standard deviation (SD).

## Figures and Tables

**Table 1:** Compositions of PUGs.

Samples	PEG/mmol				DAP /mmol	IPDI /mmol	DSO /mmol	DSO /wt%	PEG /wt%	Solid Content /wt%
	600 g/mol	1000 g/mol	1500 g/mol	2000 g/mol						
PUG–I	10	–	–	–	10	30	5.0	10.2	40.1	44.8
PUG–II	–	10	–	–	10	30	5.0	7.3	52.9	45.2
PUG–III	–	–	10	–	10	30	5.0	6.9	62.7	45.5
PUG–IV	–	–	–	10	10	30	5.0	4.4	69.1	44.8

## Data Availability

The data presented in this study are available upon request from the corresponding author.

## Supplementary Materials

The following supporting information can be downloaded at: https://www.mdpi.com/article/10.3390/gels12060555/s1. Figure S1: ^1^H NMR spectra of DSO; Figure S2: ^13^C NMR spectra of DSO; Figure S3: MS spectra of DSO.

## References

[1] Kaith B.S., Singh A., Sharma A.K., Sud D. (2021). Hydrogels: Synthesis, classification, properties and potential applications—A brief review. J. Polym. Environ..

[2] Buwalda S.J. (2024). ‘Click’ hydrogels from renewable polysaccharide resources: Bioorthogonal chemistry for the preparation of alginate, cellulose and other plant-based networks with biomedical applications. Int. J. Biol. Macromol..

[3] Wang S., Yang Q., Xu J., Zhou Y., Tian X., Wu W., Elango J., Diao X. (2025). Biofunctional carboxymethyl chitosan hydrogel incorporating hyaluronic acid and RGD peptides for accelerated wound repair. Gels.

[4] Lu P., Ruan D., Huang M., Tian M., Zhu K., Gan Z., Xiao Z. (2024). Harnessing the potential of hydrogels for advanced therapeutic applications: Current achievements and future directions. Signal Transduct. Target. Ther..

[5] Du Y., Xu Y., Hou J., Li X., Yang J., Yang J., Shan S., Wang C., Su H. (2025). Robust fabrication of pyrogallol-conjugated dextran hydrogel as antioxidant hemo-adsorbent for the selective adsorption of Pb(II). Sep. Purif. Technol..

[6] Zheng E., Zhang P., Wang J., Chen Y., Liu H., Xu J., Hou Z. (2025). Dual dynamic bonds enable biocompatible polyurethane hydrogels with superior toughness, fatigue and puncture resistance, pH-reversibility, and room-temperature self-healability. Polymer.

[7] Yang R., Xia C., Mei C., Li J. (2025). Integration of biopolymers in polyacrylic acid hydrogels: Innovations and applications in bioresources and bioproducts. J. Bioresour. Bioprod..

[8] Soliman B.G., Nguyen A.K., Gooding J.J., Kilian K.A. (2024). Advancing synthetic hydrogels through nature-inspired materials chemistry. Adv. Mater..

[9] Sepe F., Valentino A., Marcolongo L., Petillo O., Calarco A., Margarucci S., Peluso G., Conte R. (2025). Polysaccharide hydrogels as delivery platforms for natural bioactive molecules: From tissue regeneration to infection control. Gels.

[10] Maiti S., Maji B., Yadav H. (2024). Progress on green crosslinking of polysaccharide hydrogels for drug delivery and tissue engineering applications. Carbohyd. Polym..

[11] Di Martino M., Sessa L., Romano F., Piotto S., Concilio S. (2025). Tailored thermoresponsive polyurethane hydrogels: Structure–property relationships for injectable biomedical applications. Polymers.

[12] Tang Y., Wang H., Liu S., Pu L., Hu X., Ding J., Xu G., Xu W., Xiang S., Yuan Z. (2022). A review of protein hydrogels: Protein assembly mechanisms, properties, and biological applications. Colloids Surf. B Biointerfaces.

[13] Naureen B., Haseeb A.S.M.A., Basirun W.J., Muhamad F. (2021). Recent advances in tissue engineering scaffolds based on polyurethane and modified polyurethane. Mater. Sci. Eng. C.

[14] Zhang M., Xu S., Wang R., Che Y., Han C., Feng W., Wang C., Zhao W. (2023). Electrospun nanofiber/hydrogel composite materials and their tissue engineering applications. J. Mater. Sci. Technol..

[15] Divakaran A.V., Nair S.B., Karambe S.S., Wadgaonkar P.P., Nair K.S., Badiger M.V. (2025). Influence of hydrophilic/hydrophobic diols on the properties of polyurethane hydrogels: Solvent-free one-pot synthesis. J. Mater. Chem. B.

[16] Jia H.Y., Huang Z.J., Fei Z.F., Dyson P.J., Zheng Z., Wang X.L. (2016). Unconventional tough double-network hydrogels with rapid mechanical recovery, self-healing, and self-gluing properties. ACS Appl. Mater. Interfaces.

[17] Divakaran A.V., Azad L.B., Surwase S.S., Torris A.T.A., Badiger M.V. (2016). Mechanically tunable curcumin incorporated polyurethane hydrogels as potential biomaterials. Chem. Mater..

[18] Erezuma I., Lukin I., Desimone M., Zhang Y.S., Dolatshahi-Pirouz A., Orive G. (2023). Progress in self-healing hydrogels and their applications in bone tissue engineering. Biomater. Adv..

[19] Hao T., Gao Y., Zheng E., Yang H., Pan Y., Zhang P., Xu J., Hou Z. (2024). Multifunctional poly(ether-urethane) elastomer based on dynamic phenol-urethane and disulfide bonds: Simultaneously showing superior toughness, self-healing, shape memory, antibacterial, and antioxidative properties. Eur. Polym. J..

[20] Zhang K., Liu Y., Wang Z., Song C., Gao C., Wu Y. (2020). A type of self-healable, dissoluble and stretchable organosilicon elastomer for flexible electronic devices. Eur. Polym. J..

[21] Shi Z., Kang J., Zhang L. (2020). Water-enabled room-temperature self-healing and recyclable polyurea materials with super-strong strength, toughness, and large stretchability. ACS Appl. Mater. Interfaces.

[22] Li Y., He J., Luo H., He X., Liu F. (2022). Synthesis and property of room-temperature self-healable cathodic electrophoretic deposition coatings based on cationic waterborne polyurethane. J. Coat. Technol. Res..

[23] Kim S.M., Jeon H., Shin S.H., Park S.A., Jegal J., Hwang S.Y., Oh D.X., Park J. (2018). Superior toughness and fast self-healing at room temperature engineered by transparent elastomers. Adv. Mater..

[24] Mou X.Y., Yang Z.P., Lai X.J., Ding J.P., Chen Y.J., Li H.Q., Zeng X.R. (2024). Self-healing and reprocessable biobased non-isocyanate polyurethane elastomer with dual dynamic covalent adaptive network for flexible strain sensor. Chem. Eng. J..

[25] Yang B., Ding X., Xu J., Li Y., Gu R., Zhang H., Hou Z.S. (2025). Robust, self-healing polyurethane hydrogel enabled by dual crosslinking of dynamic disulfide and hydrogen bonds. Chem. J. Chin. Univ..

[26] Schwarzer L., Agarwal S. (2024). Adaptable polyurethane networks containing tertiary amines as intrinsic bond exchange catalyst. Macromol. Chem. Phys..

[27] Wu G.M., Bian J.N., Liu G.F., Chen J., Huo S.P., Jin C., Kong Z.W. (2020). Self-catalytic two-component waterborne polyurethanes with amino polyols from biomass based epoxy resin. J. Polym. Environ..

[28] Lv X., Li X.J., Zhu P.Y., Ge Y., Li Q.P., Lu H.S. (2022). Regulating redox and pH-responsive behavior of emulsion by varying alkane carbon number of tertiary amine. J. Disper. Sci. Technol..

[29] Chen Q., Zheng J., Yuan X., Wang J., Zhang L. (2018). Folic acid grafted and tertiary amino based pH-responsive pentablock polymeric micelles for targeting anticancer drug delivery. Mater. Sci. Eng. C.

[30] Jiang H., Yan T., Pang W., Cheng M., Zhao Z., He T., Wang Z., Li C., Sun S., Hu S. (2024). Incomplete ionic interactions and hydrogen bonds constructing elastomers with water accelerated Self-Healing and self-healing strengthening capacities. Chem. Eng. J..

[31] Li Y., Jin Y., Zeng W., Jin H., Shang X., Zhou R. (2023). Bioinspired fast room-temperature self-healing, robust, adhesive, and AIE fluorescent waterborne polyurethane via hierarchical hydrogen bonds and use as a strain sensor. ACS Appl. Mater. Interfaces.

[32] Wen J., Jia Z., Zhang X., Pan M., Yuan J., Zhu L. (2020). Tough, thermo-responsive, biodegradable and fast self-healing polyurethane hydrogel based on microdomain-closed dynamic bonds design. Mater. Today Commun..

[33] Kim S., Traore Y.L., Chen Y., Ho E.A., Liu S. (2018). Switchable on-demand release of a nanocarrier from a segmented reservoir type intravaginal ring filled with a pH-responsive supramolecular polyurethane hydrogel. ACS Appl. Bio Mater..

[34] Liu Y., Zhang Z., Fan W., Yang K., Li Z. (2022). Preparation of renewable gallic acid-based self-healing waterborne polyurethane with dynamic phenol–carbamate network: Toward superior mechanical properties and shape memory function. J. Mater. Sci..

[35] Fleet M.E., Harmer S.L., Liu X., Nesbitt H.W. (2005). Polarized X-ray absorption spectroscopy and XPS of TiS_3_: S K- and Ti L-ledge XANES and S and Ti 2p XPS. Surf. Sci..

[36] Qiao L., Liu C., Liu C., Zong L., Gu H., Wang C., Jian X. (2022). Self-healing, pH-sensitive and shape memory hydrogels based on acylhydrazone and hydrogen bonds. Eur. Polym. J..

[37] Zhao Z., Qin Z., Zhao T., Li Y., Hou Z., Hu H., Su X., Gao Y. (2024). Crosslinked biodegradable hybrid hydrogels based on poly(ethylene glycol) and gelatin for drug controlled release. Molecules.

[38] Ye G., Jiang T. (2021). Preparation and properties of self-healing waterborne polyurethane based on dynamic disulfide bond. Polymers.

[39] Zou F., Wang Y., Zheng Y., Xie Y., Zhang H., Chen J., Hussain M.I., Meng H., Peng J. (2022). A novel bioactive polyurethane with controlled degradation and L-Arg release used as strong adhesive tissue patch for hemostasis and promoting wound healing. Bioact. Mater..

[40] Raftopoulos K.N., Hebda E., Grzybowska A., Klonos P.A., Kyritsis A., Pielichowski K. (2022). PEG-POSS star molecules blended in polyurethane with flexible hard segments: Morphology and dynamics. Molecules.

[41] Xu J., Hao T., Liu C., Bi J., Sun J., Wen Z., Hou Z., Wei J. (2021). pH-Responsive and degradable polyurethane film with good tensile properties for drug delivery in vitro. Mater. Today Commun..

[42] Wei S., Liu J., Zhao Y., Zhang T., Zheng M., Jin F., Dong X., Xing J., Duan X. (2017). Protein-based 3D microstructures with controllable morphology and pH-responsive properties. ACS Appl. Mater. Interfaces.

[43] Deen G.R., Loh X.J. (2018). Stimuli-responsive cationic hydrogels in drug delivery applications. Gels.

[44] Tshepelevitsh S., Kutt A., Lokov M., Kaljurand I., Saame J., Heering A., Plieger P., Vianello R., Leito I. (2019). On the basicity of organic bases in different media. Eur. J. Org. Chem..

[45] Woodward P.J., Merino D.H., Greenland B.W., Hamley I.W., Light Z., Slark A.T., Hayes W. (2010). Hydrogen bonded supramolecular elastomers: Correlating hydrogen bonding strength with morphology and rheology. Macromolecules.

[46] Yilgör E., Yilgör İ. (2023). Influence of soft segment structure hydrogen bonding diisocyanate symmetry on morphology properties of segmented thermoplastic polyurethanes polyureas. Turk. J. Chem..

[47] Truong V.X., Tsang K.M., Forsythe J.S. (2017). Nonswelling click-cross-linked gelatin and PEG hydrogels with tunable properties using pluronic linkers. Biomacromolecules.

[48] Chen L., Wang S., Guo Z., Hu Y. (2022). Double dynamic bonds tough hydrogel with high self-healing properties based on acylhydrazone bonds and borate bonds. Polym. Adv. Technol..

[49] Xiang Z., Chu C., Xie H., Xiang T., Zhou S. (2021). Multifunctional thermoplastic polyurea based on the synergy of dynamic disulfide bonds and hydrogen bond cross-links. ACS Appl. Mater. Interfaces.

[50] Xu Y., Lu G., Chen M., Wang P., Li Z., Han X., Liang J., Sun Y., Fan Y., Zhang X. (2020). Redox and pH dual-responsive injectable hyaluronan hydrogels with shape-recovery and self-healing properties for protein and cell delivery. Carbohyd. Polym..

[51] Rong J., Zhong J., Yan W., Liu M., Zhang Y., Qiao Y., Fu C., Gao F., Shen L., He H. (2021). Study on waterborne self-healing polyurethane with dual dynamic units of quadruple hydrogen bonding and disulfide bonds. Polymer.

