# Supplementary material for: Robust Polyurethane Hydrogels Based on Dynamic Disulfide Bonds and Pendant Tertiary Amines with Room-Temperature Self-Healing and pH Responsiveness
Source: Gels. 2026 Jun 20;12(6):555. doi: 10.3390/gels12060555 (PMC13299447; doi:10.3390/gels12060555)
Supplement: Supplementary file 1 [file gels-12-00555-s001.zip › gels-4358633-supplementary.pdf]

Supplementary Materials

Robust Polyurethane Hydrogels Based on Dynamic Disulfide Bonds and Pendant Tertiary Amines with Room-Temperature Self-Healing and pH Responsiveness

Xia Ding [1], Bing Yang [1,*], Xinyi Si [2], Lei Ni [1], Chao Fang [2] and Zhaosheng Hou [2,*]

1 School of Intelligence Engineering, Shandong Management University, Jinan 250357, China; 14438120160212@sdmu.edu.cn (X.D.); nilch@163.com (L.N.)
2 College of Chemistry, Chemical Engineering and Materials Science, Shandong Normal University,
Jinan 250014, China; 202410100512@stu.sdnu.edu.cn (X.S.); fangchao@sdnu.edu.cn (C.F.)
* Correspondence: yangbing@sdmu.edu.cn (B.Y.); houzs@sdnu.edu.cn (Z.H.)

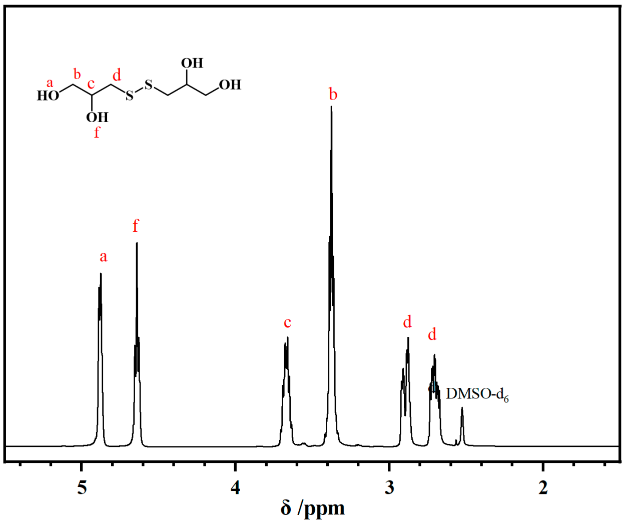

Figure S1. ^{1}HNMR spectrum of DSO.

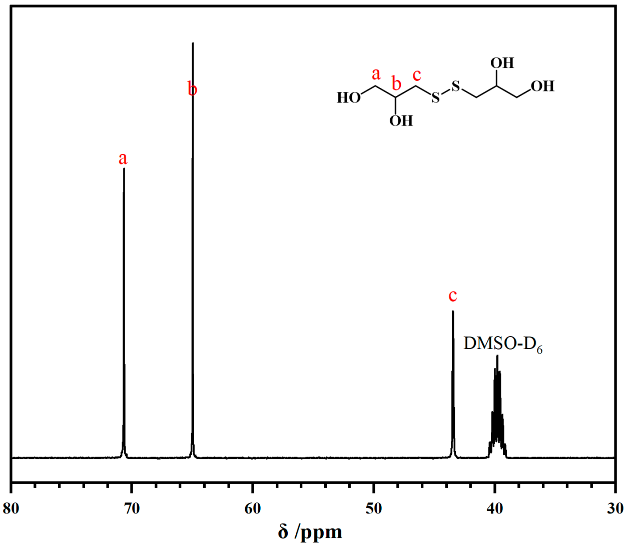

Figure S2. ^{13}C NMR spectrum of DSO.

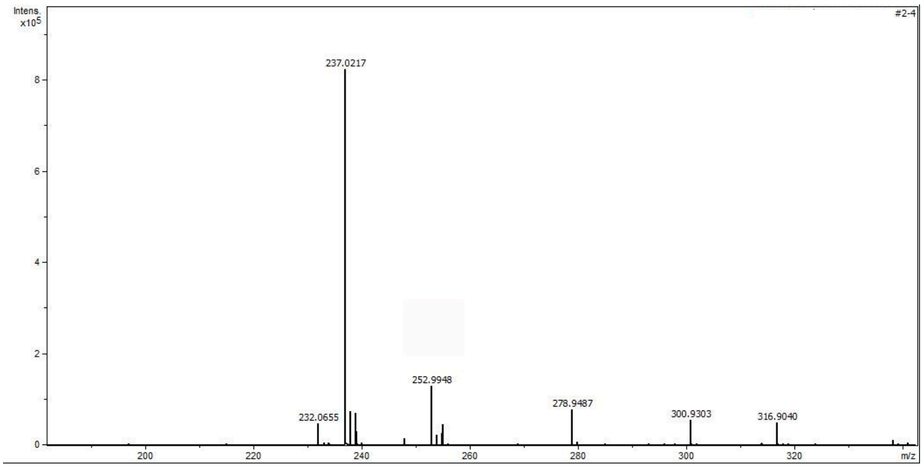

Figure S3. MS spectrum of DSO.
